# Analysis of EZH2 Genetic Variants on Triple-Negative Breast Cancer Susceptibility and Pathology

**DOI:** 10.7150/ijms.71931

**Published:** 2022-05-29

**Authors:** Liang-Chih Liu, Yi-Chung Chien, Guo-Wei Wu, Chun-Hung Hua, I-Chen Tsai, Chih-Chiang Hung, Tsai-Kun Wu, Ying-Ru Pan, Shun-Fa Yang, Yung-Luen Yu

**Affiliations:** 1 School of Medicine, College of Medicine, China Medical University, Taichung 40402, Taiwan; 2 Department of Surgery, China Medical University Hospital, Taichung 40402, Taiwan; 3 Graduate Institute of Biomedical Sciences, China Medical University, Taichung 40402, Taiwan; 4 Ph.D. Program for Translational Medicine, China Medical University, Taichung 40402, Taiwan; 5 Institute of New Drug Development, China Medical University, Taichung 40402, Taiwan; 6 Drug Development Center, Research Center for Cancer Biology, China Medical University, Taichung 40402, Taiwan; 7 Center for Molecular Medicine, China Medical University Hospital, Taichung 40402, Taiwan; 8 Department of Otorhinolaryngology Head and Neck Surgery, China Medical University Hospital, Taichung 40402, Taiwan; 9 Division of Breast Surgery, Department of Surgery, Taichung Veterans General Hospital, Taichung 40705, Taiwan; 10 Department of Applied Cosmetology, College of Human Science and Social Innovation, Hungkuang University, Taichung 43302, Taiwan; 11 Division of Renal Medicine, Tungs' Taichung MetroHarbor Hospital, Taichung 43503, Taiwan; 12 College of Medicine, National Chung Hsing University, Taichung 402204, Taiwan; 13 Department of Medical Research, Tungs' Taichung Metroharbor Hospital, Taichung, Taiwan; 14 Institute of Medicine, Chung Shan Medical University, Taichung 40201, Taiwan; 15 Department of Medical Research, Chung Shan Medical University Hospital, Taichung 40201, Taiwan; 16 Department of Medical Laboratory Science and Biotechnology, Asia University, Taichung 41354, Taiwan

**Keywords:** EZH2, triple-negative breast cancer, single-nucleotide polymorphism (SNP), age

## Abstract

Triple-negative breast cancer (TNBC) is the third most common female cancer in Taiwan. EZH2 plays an important role in cancer development through transcriptional repression by chromatin remodeling. However, the expression of EZH2 in breast cancer is highly correlated with tumorigenesis, and patient survival is not matched to TNBC. Furthermore, it has not been determined if specific EZH2 genetic variants are associated with breast cancer risk. In this paper, we evaluated the survival of different types of breast cancer. The results indicated that a lower expression of EZH2 led to poor survival of TNBC patients. Therefore, we aimed at studying the relationship between genetic polymorphisms of EZH2 and susceptibility to TNBC in Taiwan. Four single-nucleotide polymorphisms (SNPs) of EZH2 (rs6950683, rs2302427, rs3757441, and rs41277434) were analyzed by real-time PCR genotyping in 176 patients with TNBC and 1000 cancer-free controls. The results showed that TNBC patients under 60 years old who carried a TC or CC genotype at EZH2 rs6950683 and re3757441 had a tumor size of 20 mm or smaller (T1). Thus, this study is the first to examine the age and mutant genes associated with EZH2 SNPs in TNBC progression and development in Taiwan.

## 1. Introduction

Breast cancer belongs to a group of malignant tumors that grow in the breast. With the advancement of molecular medicine and pathological diagnosis, breast cancer is no longer regarded as a single disease. Furthermore, different types of breast cancer are now known to demonstrate many differences regarding the choice of clinical treatment. According to the results of a macro-array analysis of a large number of breast cancer genes, breast cancer can be divided into luminal cell type A, luminal cell type B, HER2-positive type, and basal-like cell type based on gene expression. One basal-like subtype is known as triple-negative breast cancer (TNBC). Triple-negative breast cancer refers to estrogen receptor (ER)-negative, progesterone receptor (PR)-negative, and human epidermal growth factor receptor 2 (HER-2)-negative tumors 1. This type of tumor has a worse prognosis, higher histological grade, more poorly differentiated cell types, and more aggressive characteristics and tends to relapse earlier as compared with other breast cancer subtypes 2. Although patients undergo treatment, there is still a high recurrence rate within the first 5 years, and the risk of death is relatively high, which causes patients to lose confidence in the treatment and opt to abandon further treatment 3. Therefore, the relationship between gene variants and TNBC needs to be evaluated for cancer prediction.

EZH2 is a transcriptional repressor involved in cell cycle regulation and is related to aggressive breast cancer. Compared with all other non-TNBCs, the high expression of EZH2 is closely related to the TNBC phenotype 4. EZH2 is a subunit of the multi-enzyme complex polycomb repressive complex 2 (PRC2) and functions as a histone H3 Lys27 trimethyltransferase, contributing to the epigenetic silencing of target genes and regulating the survival and metastasis of many different cancer cell types 5, 6. Studies have shown that overexpression of EZH2 is correlated with advanced stages of human cancer progression and poor prognosis 7, 8. However, we have found an inverse correlation between EZH2 and the survival of TNBC patients. Thus, we investigated the EZH2 polymorphisms and clinicopathological characteristics of TNBC.

Several recent studies have suggested that single-nucleotide polymorphisms (SNPs) are one of the most common types of genetic variation, which has an important impact on the diagnosis, treatment, and prevention of human genetic diseases 9-11. Traditionally, if a tumor suppressor gene is mutated, its tumor suppression function may be lost, thus causing cancer to occur. Moreover, when a genetic test identifies a genetic mutation, it requires careful interpretation. Some mutations are benign polymorphisms, which are unassociated with cancer; however, some are pathogenic mutations 12. Nonetheless, the effects of EZH2 polymorphisms are still unknown in TNBC. In this study, we analyzed associations among SNPs of EZH2 and TNBC susceptibility. To our knowledge, this is the first study that evaluates the EZH2 gene SNPs in triple-negative breast carcinogenesis in Taiwan.

## 2. Materials and Methods

### 2.1. Study participants and specimen collection

In this study, 176 TNBC patients were recruited from China Medical University Hospital, Taichung, Taiwan. All participants provided informed written consent during the registration process. TNBC patients were clinically staged at the time of diagnosis consistent with the tumor staging system of the American Joint Committee on Cancer (AJCC, 2002). The diagnosis of tumor staging and tumor size were collected from chart reviews. For the control group, 1000 individuals, between 20 and 70 years of age with no history of cancer, were selected from the Taiwan Biobank (https://www.twbiobank.org.tw/new_web_en/index.php). The research was approved by the Institutional Review Board of China Medical University Hospital.

### 2.2. Comprehensive Analysis of EZH2 from the Cancer Genome Atlas (TCGA)

UALCAN is a comprehensive, user-friendly, and interactive web resource for analyzing cancer OMICS data (http://ualcan.path.uab.edu/index.html). UALCAN uses TCGA level 3 RNA-seq and clinical data from 31 cancer types 13. The Kaplan-Meier plotter can assess the survival rate of the effect of 54k genes (mRNA, miRNA, protein) and 21 cancer types, including: breast (n = 7,830), ovarian (n = 2,190), lung (n = 3,452), and gastric (n = 1,440) cancer (http://kmplot.com/analysis/). Sources for the databases include GEO, EGA, and TCGA. The primary purpose of the tool is a meta-analysis-based discovery and validation of survival biomarkers 14. In this study, we used UALCAN and the KM plotter for tumor/normal differential expression analysis of EZH2 expression and overall survival analysis in different types of breast cancer patients.

### 2.3. Selection of EZH2 polymorphisms

For this study, four SNPs in EZH2 (NM_004456) were selected from the International HapMap Project data. We included the non-synonymous SNP rs2302427 (D185H in exon 6) in the coding sequence of the gene. The other SNPs (rs6950683, rs3757441, and rs41277434) were selected in this study since they have been found in cancer patients.

### 2.4. Genomic DNA extraction

We extracted genomic DNA using the QIAamp DNA Blood Mini Kit Reagent (Qiagen, Valencia, CA). DNA was dissolved in buffer with 10 mM Tris (pH 7.8) and 1 mM EDTA. Then, DNA quantification was measured at an optical density of 260 nm. The final DNA preparations were stored at -20 °C and used as templates for PCR.

### 2.5. Real-time PCR

Allelic discrimination of the EZH2 polymorphisms rs6950683, rs2302427, rs3757441, and rs41277434 was assessed using the ABI StepOneTM Real-Time PCR System (Applied Biosystems), SDS v3.0 software (Applied Biosystems), and the TaqMan assay. The final volume for each reaction was 5 μL, containing 2.5 μL TaqMan Genotyping Master Mix, 0.125 μL TaqMan probes mix, and 10 ng genomic DNA. The reaction conditions included an initial denaturation step at 95 °C for 10 min followed by 40 cycles at 95 °C for 15 sec and 60 °C for 1 min.

### 2.6. Statistical analysis

The differences in age and demographic characteristics between control and OSCC patients were compared by the Mann-Whitney* U*-test. The odds ratios (ORs) with 95% confidence intervals (CIs) were estimated by logistic regression models. After controlling for other covariates, the adjusted odds ratios (AORs) with 95% CIs of the association between genotype frequencies, OSCC risk, and clinicopathological characteristics were estimated by multiple logistic regression models. Values of *p* < 0.05 were considered significant. All the data were analyzed using SAS statistical software (Version 9.1, 2005; SAS Institute Inc., Cary, NC).

## 3. Results

**Figure 1:**
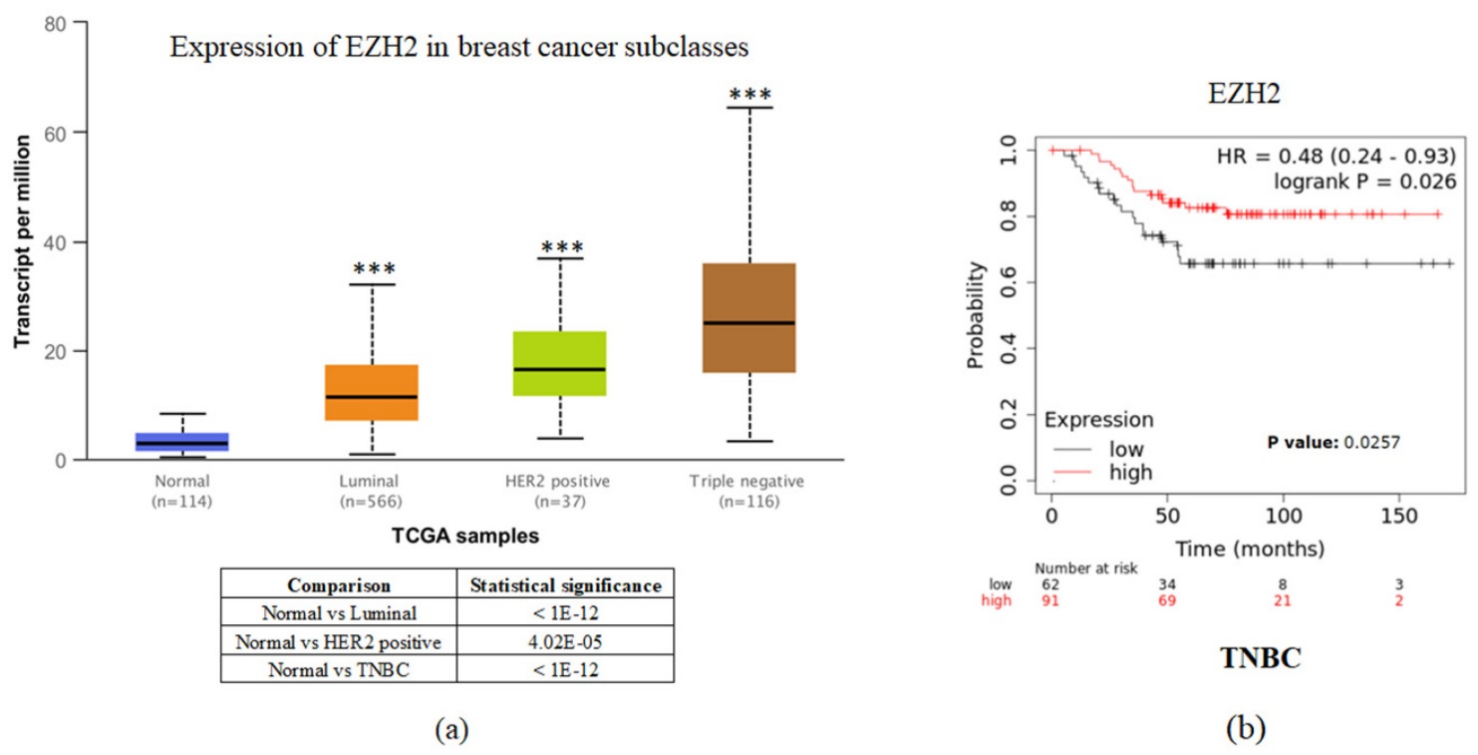
*EZH2* expression is correlated with breast cancer progression but not with the survival rate of different breast cancer subtypes. (a) Expression of *EZH2* in normal control and different subtypes of breast cancer patients as assessed with data from UALCAN. (b) Correlation between different levels of *EZH2* in TNBC patients as assessed with data from KM plotter.

To investigate the clinical impact of EZH2 on breast cancer progression, we used UALCAN and the Kaplan-Meier plotter to assess the relationship between cellular levels of *EZH2* mRNA and different types of breast cancer patient outcomes. All breast cancer patients with higher *EZH2* expression compared to the normal control had significant results (Figure 1a). Interestingly, inconsistent with the general results of EZH2 for triple-negative breast cancer, we found that patients with lower *EZH2* expression had significantly shorter overall survival than those with higher *EZH2* expression in TNBC patients (Figure 1b). This result implies that the regulation of EZH2 in TNBC may have unknown mechanisms.

In order to find out the possible cause of triple-negative breast cancer in a clinical setting, for this case-cohort study, a total of 1000 healthy controls and 176 patients with TNBC were recruited. According to our age analysis of TNBC patients (Table 1), we found significant differences in age (*p* < 0.001) between patients with TNBC and healthy groups.

Table 2 shows the distribution frequency of *EZH2* genotypes in controls and patients with TNBC. No significant differences with respect to rs6950683, rs2302427, rs3757441, and rs41277434 polymorphisms of *EZH2* were observed between healthy controls and patients with TNBC (Table 2).

Next, we further evaluated the effect of the polymorphic genotypes of EZH2 (rs6950683, rs2302427, rs3757441, and rs41277434) on the clinical status of TNBC (Table 3). As shown in Table 3, patients with at least one polymorphic C allele at the rs6950683 and rs3757441 SNPs (TC + CC genotype) seem to have some differences in tumor size performance but without significant difference.

Therefore, according to the results of Table 1—which indicated significant differences in age between patients with TNBC and healthy groups—we further evaluated the effect of the EZH2 polymorphic genotypes (rs6950683, rs2302427, rs3757441, and rs41277434) on the clinical status of TNBC under 60 years old (Table 4). Furthermore, the results indicated that there are significant differences in rs6950683 and rs3757441 SNPs (TC + CC genotype) with tumor size. These results indicated that *EZH2* polymorphisms have a great influence and significant difference on triple-negative breast cancer patients under 60 years old.

## 4. Discussion

EZH2 is a part of PRC2, which plays an important role in the epigenetic regulation of gene expression, and regulates cancer cells proliferation, migration, invasion, and stemness and functions as an oncogenic factor in most solid tumors 15-17. Women under 60 years are more likely to present with aggressive subtypes such as TNBC, and HER2-positive breast cancer, whereas older women have higher rates of luminal A subtype breast cancer 18. Age-related differences in tumor biology, treatment, and outcomes have yet to be fully characterized within TNBC. Moreover, EZH2 is frequently overexpressed in many cancer types and is associated with a poor prognosis 19-22. Interestingly, in this study, in TNBC patients, lower EZH2 expression had significantly shorter overall survival than higher EZH2 expression (Figure 1b). In Table 1, significant differences in age between patients with TNBC and healthy groups were observed. Therefore, this is the first study to evaluate the influence of age on tumor size and gene mutation in a TNBC patient population. However, according to our results, several issues remain to be discussed.

SNP is a nucleotide variation that occurs at the DNA level of all human cells. Related to environmental factors, SNPs can mimic the diversity of human phenotypes and can also indicate susceptibility to various diseases, including cancer 23. In order to distinguish the effects of SNP from other types of genetic mutations, the incidence of each polymorphism must be greater than the incidence of a single natural mutation. Changes in the function of gene products—due to mutations or genetic polymorphisms—may lead to increased cancer risk and certain disease phenotypes 24. Indeed, several papers regarding the EZH2 SNPs and cancer progression have been published. In cholangiocarcinoma, rs887569 EZH2 SNP may serve as a possible predictive marker of overall survival in advanced CCA patients 25. It is also repoted that the SNPs of *EZH2* and *DNMT1* are risk predictors for TNBC, but only the T allele of rs2288349 and the C allele of rs16999593 of *DNMT1* increase the risk of TNBC. However, the result only indicated that the G allele of rs10274701 of *EZH2* significantly increased the expression level of EZH2 in TNBC 26. Furthermore, 12 eligible studies have indicated that rs887569 and rs2302427 in *EZH2* may be correlated with a decreased cancer risk. Moreover, rs3757441 and rs41277434 are independent risk factors of cancer. However, further large-scale and functional studies are warranted to validate these findings 27.

In this study, we found that EZH2 rs6950683 and rs3757441 SNPs (TC + CC genotype) are related to the tumor size of TNBC patients under 60 years old. In our previous study, we found that these two EZH2 SNPs (rs6950683 and rs3757441) might contribute to the prediction of oral squamous cell carcinoma (OSCC) susceptibility. Moreover, the rs6950683 CC genotype exhibits hypermethylation in the EZH2 promoter and decreases OSCC susceptibility 28. In summary, these findings require additional functional studies for further confirmation.

## 5. Conclusions

The findings of this study suggest that the interaction of clinical features of genes may alter the susceptibility to TNBC. Importantly, this study provides new information regarding the relationship between EZH2 polymorphisms and TNBC clinical pathology in the Taiwanese population.

## Figures and Tables

**Table 1:** Distributions of demographical characteristics in 1000 controls and 176 patients with breast cancer.

Variable	Controls (N=1000)	Patients (N=176)	*p*-Value
Age (yrs)	Mean ± S.D.	Mean ± S.D.	
	56.27 ± 11.09	61.19 ± 12.66	<0.001*
Tumor T status			
<T2		119 (67.6%)	
≥T2		57 (32.4%)	
			

Fisher's exact test was used between healthy controls and patients with breast cancer. *, *p*-value < 0.05 is statistically significant.

**Table 2:** Distribution frequency of *EZH2* genotypes in 1000 healthy controls and 176 patients with breast cancer.

Variable	Controls (N=1000) n (%)	Patients (N=176) n (%)	OR (95% CI)	AOR (95% CI)
rs6950683				
TT	560 (56.0%)	102 (58.0%)	1.00	1.00
TC	389 (38.9%)	67 (38.1%)	0.946 (0.677-1.321)	1.057 (0.706-1.583)
CC	51 (5.1%)	7 (4.0%)	0.754 (0.333-1.707)	0.452 (0.152-1.346)
TC+CC	440 (44.0%)	74 (42.0%)	0.923 (0.668-1.277)	0.963 (0.651-1.423)
rs2302427				
CC	623 (62.3%)	116 (65.9%)	1.00	1.00
CG	336 (33.6%)	57 (32.4%)	0.911 (0.646-1.285)	1.121 (0.744-1.688)
GG	41 (4.1%)	3 (1.7%)	0.393 (0.120-1.290)	0.441 (0.101-1.928)
CG+GG	377 (37.7%)	60 (34.1%)	0.855 (0.610-1.197)	1.052 (0.703-1.573)
rs3757441				
TT	559 (55.9%)	101 (57.4%)	1.00	1.00
TC	388 (38.8%)	68 (38.6%)	0.970 (0.695-1.354)	1.109 (0.741-1.659)
CC	53 (5.3%)	7 (4.0%)	0.731 (0.323-1.653)	0.443 (0.149-1.315)
TC+CC	441 (44.1%)	75 (42.6%)	0.941 (0.681-1.301)	1.002 (0.678-1.479)
rs41277434				
AA	949 (94.9%)	164 (93.2%)	1.00	1.00
AC	51 (5.1%)	12 (6.8%)	1.362 (0.711-2.609)	0.963 (0.359-2.580)
CC	0 (0%)	0 (0%)	----	----
AC+CC	51 (5.1%)	12 (6.8%)	1.362 (0.711-2.609)	0.963 (0.359-2.580)

Odds ratios (ORs) and their 95% confidence intervals (CIs) were estimated by logistic regression models. Adjusted odds ratios (AORs) with their 95% confidence intervals (CIs) were estimated by multiple logistic regression models after controlling for age.

**Table 3:** Adjusted odds ratio (AOR) and 95% confidence interval (CI) of clinical status and *EZH2* genotypic frequencies in 176 breast cancer patients.

Variable	Tumor Size			
	<T2 (N=119)	≥T2 (N=57)	OR (95% CI)	*p*-Value
EZH2 SNP				
rs6950683				
TT	63 (52.9%)	39 (68.4%)	1.00	0.052
TC+CC	56 (47.1%)	18 (31.6%)	0.519 (0.267-1.009)	
rs2302427				
CC	78 (65.5%)	38 (66.7%)	1.00	0.883
CG+GG	41 (34.5%)	19 (33.3%)	0.951 (0.488-1.855)	
rs3757441				
TT	63 (52.9%)	38 (66.7%)	1.00	0.085
TC+CC	56 (47.1%)	19 (33.3%)	0.563 (0.291-1.086)	
rs41277434				
AA	111 (93.3%)	53 (93.0%)	1.00	0.942
AC+CC	8 (6.7%)	4 (7.0%)	1.047 (0.302-3.633)	

ORs analyzed by their 95% CIs were estimated by logistic regression models.

**Table 4:** Adjusted odds ratio (AOR) and 95% confidence interval (CI) of clinical status and *EZH2* genotypic frequencies in 80 breast cancer patients with age below 60.

Variable	Tumor size			
	<T2 (N=51)	≥ T2 (N=29)	OR (95% CI)	*p*-Value
EZH2 SNP				
rs6950683				
TT	27 (52.9%)	23 (79.3%)	1.00	0.019*
TC+CC	24 (47.1%)	6 (20.7%)	0.293 (0.102-0.841)	
rs2302427				
CC	33 (64.7%)	17 (58.6%)	1.00	0.589
CG+GG	18 (35.3%)	12 (41.4%)	1.294 (0.508-3.299)	
rs3757441				
TT	27 (52.9%)	22 (75.9%)	1.00	0.043*
TC+CC	24 (47.1%)	7 (24.1%)	0.358 (0.130-0.986)	
rs41277434				
AA	51 (100%)	29 (100%)	1.00	---
AC+CC	0 (0%)	0 (0%)	---	

ORs analyzed by their 95% CIs were estimated by logistic regression models. * *p*-value < 0.05 is statistically significant.

## References

[1] Blenkiron C, Goldstein LD, Thorne NP, Spiteri I, Chin SF, Dunning MJ (2007). MicroRNA expression profiling of human breast cancer identifies new markers of tumor subtype. Genome Biol.

[2] Lehmann BD, Pietenpol JA (2014). Identification and use of biomarkers in treatment strategies for triple-negative breast cancer subtypes. J Pathol.

[3] Liedtke C, Mazouni C, Hess KR, Andre F, Tordai A, Mejia JA (2008). Response to neoadjuvant therapy and long-term survival in patients with triple-negative breast cancer. J Clin Oncol.

[4] Hussein YR, Sood AK, Bandyopadhyay S, Albashiti B, Semaan A, Nahleh Z (2012). Clinical and biological relevance of enhancer of zeste homolog 2 in triple-negative breast cancer. Hum Pathol.

[5] Chang CJ, Hung MC (2012). The role of EZH2 in tumour progression. Br J Cancer.

[6] Schuettengruber B, Chourrout D, Vervoort M, Leblanc B, Cavalli G (2007). Genome regulation by polycomb and trithorax proteins. Cell.

[7] Chien YC, Liu LC, Ye HY, Wu JY, Yu YL (2018). EZH2 promotes migration and invasion of triple-negative breast cancer cells via regulating TIMP2-MMP-2/-9 pathway. Am J Cancer Res.

[8] Varambally S, Cao Q, Mani RS, Shankar S, Wang X, Ateeq B (2008). Genomic loss of microRNA-101 leads to overexpression of histone methyltransferase EZH2 in cancer. Science.

[9] Chuang CY, Chien YC, Lin CW, Chou CH, Chen SC, Liu CL (2021). TRIM21 Polymorphisms are associated with Susceptibility and Clinical Status of Oral Squamous Cell Carcinoma patients. Int J Med Sci.

[10] Kwok PY, Chen X (2003). Detection of single nucleotide polymorphisms. Curr Issues Mol Biol.

[11] Wang DG, Fan JB, Siao CJ, Berno A, Young P, Sapolsky R (1998). Large-scale identification, mapping, and genotyping of single-nucleotide polymorphisms in the human genome. Science.

[12] Spurdle AB, Healey S, Devereau A, Hogervorst FB, Monteiro AN, Nathanson KL (2012). ENIGMA-evidence-based network for the interpretation of germline mutant alleles: an international initiative to evaluate risk and clinical significance associated with sequence variation in BRCA1 and BRCA2 genes. Hum Mutat.

[13] Chandrashekar DS, Bashel B, Balasubramanya SAH, Creighton CJ, Ponce-Rodriguez I, Chakravarthi B (2017). UALCAN: A Portal for Facilitating Tumor Subgroup Gene Expression and Survival Analyses. Neoplasia.

[14] From the American Association of Neurological Surgeons ASoNC, Interventional Radiology Society of Europe CIRACoNSESoMINTESoNESOSfCA, Interventions SoIRSoNS, World Stroke O, Sacks D, Baxter B, et al (2018). Multisociety Consensus Quality Improvement Revised Consensus Statement for Endovascular Therapy of Acute Ischemic Stroke. Int J Stroke.

[15] Chang LC, Yu YL (2016). Dietary components as epigenetic-regulating agents against cancer. Biomedicine (Taipei).

[16] Donaldson-Collier MC, Sungalee S, Zufferey M, Tavernari D, Katanayeva N, Battistello E (2019). EZH2 oncogenic mutations drive epigenetic, transcriptional, and structural changes within chromatin domains. Nat Genet.

[17] Svedlund J, Barazeghi E, Stalberg P, Hellman P, Akerstrom G, Bjorklund P (2014). The histone methyltransferase EZH2, an oncogene common to benign and malignant parathyroid tumors. Endocr Relat Cancer.

[18] Howlader N, Cronin KA, Kurian AW, Andridge R (2018). Differences in Breast Cancer Survival by Molecular Subtypes in the United States. Cancer Epidemiol Biomarkers Prev.

[19] Chang WS, Tsai CW, Yang JS, Hsu YM, Shih LC, Chiu HY (2021). Resveratrol inhibited the metastatic behaviors of cisplatin-resistant human oral cancer cells via phosphorylation of ERK/p-38 and suppression of MMP-2/9. J Food Biochem.

[20] Liu SP, Shibu MA, Tsai FJ, Hsu YM, Tsai CH, Chung JG (2020). Tetramethylpyrazine reverses high-glucose induced hypoxic effects by negatively regulating HIF-1alpha induced BNIP3 expression to ameliorate H9c2 cardiomyoblast apoptosis. Nutr Metab (Lond).

[21] Xia L, Zhu X, Zhang L, Xu Y, Chen G, Luo J (2020). EZH2 enhances expression of CCL5 to promote recruitment of macrophages and invasion in lung cancer. Biotechnol Appl Biochem.

[22] Zheng N, Chu M, Lin M, He Y, Wang Z (2020). USP7 stabilizes EZH2 and enhances cancer malignant progression. Am J Cancer Res.

[23] Dong LM, Potter JD, White E, Ulrich CM, Cardon LR, Peters U (2008). Genetic susceptibility to cancer: the role of polymorphisms in candidate genes. JAMA.

[24] Yu YL, Su KJ, Hsieh YH, Lee HL, Chen TY, Hsiao PC (2013). Effects of EZH2 polymorphisms on susceptibility to and pathological development of hepatocellular carcinoma. PLoS One.

[25] Paolicchi E, Pacetti P, Giovannetti E, Mambrini A, Orlandi M, Crea F (2013). A single nucleotide polymorphism in EZH2 predicts overall survival rate in patients with cholangiocarcinoma. Oncol Lett.

[26] Tao R, Chen Z, Wu P, Liu C, Peng Y, Zhao W (2015). The possible role of EZH2 and DNMT1 polymorphisms in sporadic triple-negative breast carcinoma in southern Chinese females. Tumour Biol.

[27] Ling Z, You Z, Hu L, Zhang L, Wang Y, Zhang M (2018). Effects of four single nucleotide polymorphisms of EZH2 on cancer risk: a systematic review and meta-analysis. Onco Targets Ther.

[28] Su KJ, Lin CW, Chen MK, Yang SF, Yu YL (2015). Effects of EZH2 promoter polymorphisms and methylation status on oral squamous cell carcinoma susceptibility and pathology. Am J Cancer Res.

